# On the Fourth Power Mean of the Two-Term Exponential Sums

**DOI:** 10.1155/2014/724840

**Published:** 2014-01-12

**Authors:** Han Zhang, Wenpeng Zhang

**Affiliations:** Department of Mathematics, Northwest University, Xi'an, Shaanxi, China

## Abstract

The main purpose of this paper is to use the analytic methods and the properties of Gauss sums to study the computational problem of one kind fourth power mean of two-term exponential sums and give an interesting identity and asymptotic formula for it.

## 1. Introduction

Let *q* ≥ 3 be a positive integer. For any integers *m* and *n*, the two-term exponential sum *C*(*m*, *n*, *k*; *q*) is defined as follows:

(1) $$\begin{array}{c} C\left(m,n,k;q\right)=\overset{q}{\underset{a=1}{\sum }}e\left(\frac{ma^{k}+na}{q}\right), \end{array}$$

where *e*(*y*) = *e* ^2*πiy*^.

Regarding the various properties of *C*(*m*, *n*, *k*; *q*), some authors have studied them, and obtained a series of results. Some related works can be found in references [1–8]. For example, Gauss' classical work proved the remarkable formula (see [1])

(2) C(1,0,2;q)=12q(1+i)(1+e(−q4))={q,if  q≡1mod4,0,if  q≡2mod4,iq,if  q≡3mod4,(1+i)q,if  q≡0mod4,

where *i* ^2^ = −1.

Generally, for any odd number *q* and (*m*, *q*) = 1, the exact value of |*C*(*m*, 0,2; *q*)| is $\sqrt{q}$, More relevant to it, Cochrane and Zheng [4] showed the general sum that

(3) $$\begin{array}{c} \left|C\left(m,n,k;q\right)\right|\leq k^{\omega (q)}q^{1/2}, \end{array}$$

where *ω*(*q*) denotes the number of all distinct prime divisors of *q*.

In this paper, we study the fourth power mean of the two-term exponential sum *C*(*m*, *n*, *k*; *q*) as follows:

(4) $$\begin{array}{c} \overset{q}{\underset{m=1}{\sum }}{\left|C\left(m,n,k;q\right)\right|}^{4}, \end{array}$$

where *n* is any integer with (*n*, *q*) = 1.

Regarding this problem, it seems that none has studied it yet; at least we have not seen any related result before. The problem is interesting, because it can reflect that the mean value of *C*(*m*, *n*, *k*; *q*) is well behaved. The main purpose of this paper is to use the analytic methods and the properties of Gauss sums to study the special case of (4) with *k* = 5, *q* = *p*, an odd prime and give an interesting identity and asymptotic formula for it. That is, we will prove the following conclusion.

**Theorem 1**

Let *p* > 3 be a prime. Then for any integer *n* with (*n*, *p*) = 1, one has the identity

(5) ∑m=1p|∑a=1p−1e(ma5+nap)|4 ={3p3−p2(8+2(−1p)+4(−3p))−3p,if  5†p−1,3p3+O(p2),if  5 ∣ p−1,

where (∗/*p*) denotes the Legendre symbol.

For any prime *p* with 5 | *p* − 1, one cannot give an exact computational formula in our theorem at present. The difficulty is that one needs to know the value of the character sums

(6) $$\begin{array}{c} \overset{4}{\underset{i=1}{\sum }}{\left|\overset{p-1}{\underset{a=1}{\sum }}\chi_{1}^{i}\left(a^{5}-1\right)\right|}^{2}, \end{array}$$

where *χ*_1_ is any 5-order character mod *p*.

For any integer *h* ≥ 3, whether there exists an exact computational formula for

(7) $$\begin{array}{c} \overset{p}{\underset{m=1}{\sum }}{\left|\overset{p-1}{\underset{a=1}{\sum }}e\left(\frac{ma^{5}+na}{p}\right)\right|}^{2h} \end{array}$$

is an open problem, where *p* is an odd prime and (*n*, *p*) = 1.

## 2. Several Lemmas

In this section, we will give several lemmas which are necessary in the proof of our theorem. In the proving process of all lemmas, we used many properties of Gauss sums; all these can be found in [1], so they will not be repeated here. First we have the following.

**Lemma 2**

Letting *p* be an odd prime with *p* > 3, then one has the identity

(8) $$\begin{array}{c} \underset{p\;|\;\left(a-b\right)\left(a+b+1\right)\left(a^{2}+b^{2}+a+b+1\right)}{\overset{p-2}{\underset{a=1}{\sum }}\;\overset{p-2}{\underset{b=1}{\sum }}}1=3p-10-2\left(\frac{-1}{p}\right)-4\left(\frac{-3}{p}\right), \end{array}$$

where (∗/*p*) denotes the Legendre's symbol.

**Proof**

For any prime *p*, note that if *a* passes through a complete residue system mod *p*, then 2*a* + 1 also passes through a complete residue system mod *p*, so note the identity

(9) $$\begin{array}{c} \overset{p}{\underset{a=1}{\sum }}\left(\frac{a^{2}+n}{p}\right)=\{\begin{array}{cc} -1, & \text{if}\;\left(n,p\right)=1,\\ p-1, & \text{if}\;\left(n,p\right)=p \end{array} \end{array}$$

(this formula can be found in Hua's book, Section 7.8, Theorem 8.2 [9]). One has

(10) ∑a=1p−2 ∑b=1p−2p ∣ (a−b)(a+b+1)(a2+b2+a+b+1)1 =∑a=1p−2 ∑b=1p−2p ∣ (a−b)(a+b+1)p†a2+b2+a+b+11+∑a=1p−2 ∑b=1p−2p†(a−b)(a+b+1)p ∣ a2+b2+a+b+11+∑a=1p−2 ∑b=1p−2p ∣ (a−b)(a+b+1)p ∣ a2+b2+a+b+11 =2(p−2)−1−∑a=1p−2p ∣ 2a2+2a+11+∑a=1p−2 ∑b=1p−2p ∣ a2+b2+a+b+11 =2p−5−∑a=1p−2p ∣ (2a+1)2+11−4∑a=0p−1p ∣ (2a+1)2+31  +∑a=0p−1 ∑b=0p−1p ∣ (2a+1)2+(2b+1)2+21 =2p−5−∑a=0p−1p ∣ a2+11−4∑a=0p−1p ∣ a2+31+∑a=0p−1 ∑b=0p−1p ∣ a2+b2+21 =2p−5−(1+(−1p))−4(1+(−3p))  +∑b=0p−1(1+(−(b2+2)p)) =3p+∑b=0p−1(−(b2+2)p)−10−4(−3p)−(−1p) =3p−10−2(−1p)−4(−3p).

This proves Lemma 2.

**Lemma 3**

Let *p* be an odd prime *χ* be any nonprincipal character mod *p*. Then for any integer *n* with (*n*, *p*) = 1, one has the identity

(11) |∑m=1p−1χ(m)|∑a=1p−1e(ma5+nap)|2| ={p|∑a=1p−2χ¯(5a4+10a3+10a2+5a+1)|,  if χ is not a 5-order character modp,p|−2+2∑a=1p−1χ¯(a(1−a))+∑a=1p−1χ¯(a2(a−1))|,  if χ is a 5-order character modp.

**Proof**

Note that *χ* is a non-principal character mod *p*, so if *χ* is not a 5-order character mod *p*, (i.e., *χ* ^5^ ≠ *χ*_0_, the principal character mod *p*), then from the properties of Gauss sums we have

(12) ∑m=1p−1χ(m)|∑a=1p−1e(ma5+nap)|2 =∑a=1p−1 ∑b=1p−1 ∑m=1p−1χ(m)e(m(a5−b5)+n(a−b)p) =∑a=1p−1 ∑b=1p−1 ∑m=1p−1χ(m)e(mb5(a5−1)+nb(a−1)p) =τ(χ)∑a=1p−1χ¯(a5−1)∑b=1p−1χ¯5(b)e(nb(a−1)p) =τ(χ)τ(χ¯5)∑a=1p−1χ¯(a5−1)χ5(n(a−1)) =χ5(n)τ(χ)τ(χ¯5)∑a=1p−2χ¯((a+1)5−1)χ(a5) =χ5(n)τ(χ)τ(χ¯5)∑a=1p−2χ¯((a¯+1)5−a¯5) =χ5(n)τ(χ)τ(χ¯5)∑a=1p−2χ¯(5a4+10a3+10a2+5a+1),

where *τ*(*χ*) = ∑_*a*=1_^*p*−1^ *χ*(*a*)*e*(*a*/*p*) denotes the classical Gauss sums.

If *χ* is a 5-order character mod *p*, then *χ* ^5^ = *χ*_0_; note that for any integer *a* with (*a*, *p*) = 1, we have $\chi^{4}(a)=\bar{\chi }(a)$, $\chi^{3}(a)={\bar{\chi }}^{2}(a)$, *χ*(−1) = −1, and

(13) χ4(a)+χ3(a)+χ2(a)+χ(a)+1 ={5,if a is a 5th residue modp,0,otherwise.

From the method of proving (12) we have the identity

(14) ∑m=1p−1χ(m)|∑a=1p−1e(ma5+nap)|2 =τ(χ)∑a=1p−1χ¯(a5−1)∑b=1p−1χ¯5(b)e(nb(a−1)p) =τ(χ)∑a=1p−1χ¯(a5−1)∑b=1p−1e(nb(a−1)p) =−τ(χ)∑a=1p−1χ¯(a5−1) =−τ(χ)∑a=1p−1(χ4(a)+χ3(a)+χ2(a)+χ(a)+1)χ¯(a−1) =−τ(χ)(2∑a=1p−1χ¯(a−1)+2∑a=1p−1χ¯(a¯(1−a¯))       +∑a=1p−1χ¯(a2(a−1))) =−τ(χ)(−2+2∑a=1p−1χ¯(a(1−a))+∑a=1p−1χ¯(a2(a−1))).

Now note that $\left|\tau \left(\chi \right)\right|=\sqrt{p}$ if *χ* ≠ *χ*_0_. From (12) and (14) we may immediately deduce Lemma 3.

**Lemma 4**

Let *p* be an odd prime and let *χ* be a 5th character mod *p*. Then one has the identity

(15) $$\begin{array}{c} \overset{p-1}{\underset{a=1}{\sum }}\chi \left(a\left(a-1\right)\right)=\frac{\tau^{2}\left(\chi \right)}{\tau \left(\chi^{2}\right)},\\ \overset{p-1}{\underset{a=1}{\sum }}\chi \left(a^{2}\left(a-1\right)\right)=\frac{\tau \left(\chi \right)\tau \left(\chi^{2}\right)}{\tau \left(\chi^{3}\right)}. \end{array}$$

Therefore

(16) $$\begin{array}{c} \left|\overset{p-1}{\underset{a=1}{\sum }}\chi \left(a\left(a-1\right)\right)\right|=\sqrt{p},\;\;\left|\overset{p-1}{\underset{a=1}{\sum }}\chi \left(a^{2}\left(a-1\right)\right)\right|=\sqrt{p}. \end{array}$$

**Proof**

Noting that *χ*(−1) = 1, from the definition and properties of the classical Gauss sums, we have

(17) τ2(χ)=∑a=1p−1 ∑b=1p−1χ(a)χ(b)e(a+bp)=∑a=1p−1χ(a)∑b=1p−1χ2(b)e(b(a+1)p)=τ(χ2)∑a=1p−1χ(a)χ¯((a+1)2)=τ(χ2)∑a=2pχ(a−1)χ¯(a2)=τ(χ2)∑a=2p−1χ((1−a¯)a¯)=τ(χ2)∑a=1p−1χ(a(1−a))

or

(18) $$\begin{array}{c} \overset{p-1}{\underset{a=1}{\sum }}\chi \left(a\left(a-1\right)\right)=\frac{\tau^{2}\left(\chi \right)}{\tau \left(\chi^{2}\right)}. \end{array}$$

Similarly, we also have

(19) τ(χ)τ(χ2)=∑a=1p−1 ∑b=1p−1χ(a)χ2(b)e(a+bp)=∑a=1p−1χ(a)∑b=1p−1χ3(b)e(b(a+1)p)=τ(χ3)∑a=1p−1χ(a)χ¯((a+1)3)=τ(χ3)∑a=2pχ(a−1)χ¯(a3)=τ(χ3)∑a=2p−1χ((1−a¯)a¯2)=τ(χ3)∑a=1p−1χ(a2(1−a))

or

(20) $$\begin{array}{c} \overset{p-1}{\underset{a=1}{\sum }}\chi \left(a^{2}\left(a-1\right)\right)=\frac{\tau \left(\chi \right)\tau \left(\chi^{2}\right)}{\tau \left(\chi^{3}\right)}. \end{array}$$

This proves Lemma 4.

## 3. Proof of the Theorem

In this section, we shall complete the proof of our theorem. First from the orthogonality of characters mod *p* we have

(21) ∑χmodp|∑m=1p−1χ(m)|∑a=1p−1e(ma5+nap)|2|2 =(p−1)∑m=1p−1|∑a=1p−1e(ma5+nap)|4.

On the other hand, if 5†*p* − 1, then any non-principal character *χ* is not a 5-order character mod *p*. Note that

(22) ∑m=1p−1χ0(m)|∑a=1p−1e(ma5+nap)|2 =∑a=1p−1 ∑b=1p−1 ∑m=1p−1e(mb5(a5−1)+nb(a−1)p) =(p−1)2+p−2=p2−p−1.

From (22) and Lemma 3 we have

(23) ∑χmodp|∑m=1p−1χ(m)|∑a=1p−1e(ma5+nap)|2|2 =|∑m=1p−1χ0(m)|∑a=1p−1e(ma5+nap)|2|2  +∑χmodpχ≠χ0|∑m=1p−1χ(m)|∑a=1p−1e(ma5+nap)|2|2 =(p2−p−1)2  +∑χmodpχ≠χ0p2·|∑a=1p−2χ¯(5a4+10a3+10a2+5a+1)|2 =(p2−p−1)2  +p2∑χmodp|∑a=1p−2χ¯(5a4+10a3+10a2+5a+1)|2  −p2(∑a=1p−2χ0(5a4+10a3+10a2+5a+1))2 =(p2−p−1)2+p2(p−1)  ×∑a=1p−2 ∑b=1p−2a4+2a3+2a2+a≡b4+2b3+2b2+bmodp1−p2(p−2)2 =(p2−p−1)2+p2(p−1)  ×∑a=1p−2 ∑b=1p−2(a−b)(a+b+1)(a2+b2+a+b+1)≡0modp1−p2(p−2)2 =(p2−p−1)2+p2(p−1)  ×(3p−10−2(−1p)−4(−3p))−p2(p−2)2 =(p−1)[3p3−p2(8+2(−1p)+4(−3p))−3p−1].

If 5†*p* − 1, then combining (21) and (23) we may immediately deduce the identity

(24) ∑m=1p−1|∑a=1p−1e(ma5+nap)|4 =3p3−p2(8+2(−1p)+4(−3p))−3p−1

or

(25) ∑m=1p|∑a=1p−1e(ma5+nap)|4 =3p3−p2(8+2(−1p)+4(−3p))−3p.

If 5 | *p* − 1, since *χ*_1_ ≠ *χ*_0_ is a 5-order character mod *p*, $\bar{\chi_{1}}=\chi_{1}^{4}$ and ${\bar{\chi_{1}}}^{2}=\chi_{1}^{3}$ are also 5-order characters mod *p*, then note that

(26) ∑m=1p−1χ0(m)|∑a=1p−1e(ma5+nap)|2 =(p−1)2−4(p−1)+p−6=p2−5p−1∑a=1p−2χ1((a+1)5−a5) =∑a=1p−2χ1((a¯+1)5−1)=∑a=1p−1χ1(a5−1) =∑a=1p−1(χ12(a)+χ1(a)+χ1¯2(a)+χ1¯(a)+1)χ1(a−1) =∑a=1p−1χ1(a2(1−a))+2∑a=1p−1χ1(a(1−a))−2.

From Lemma 4 and the method of proving (23) we have

(27) ∑χmodp|∑m=1p−1χ(m)|∑a=1p−1e(ma5+nap)|2|2 =(p2−5p−1)2  +∑χmodpχ≠χ0,χ1,χ12,χ13,χ14p2|∑a=1p−2χ¯(5a4+10a3+10a2+5a+1)|2  +p∑i=14|∑a=1p−1χ1i(a2(1−a))+2∑a=1p−1χ1i(a(1−a))−2|2 =(p2−5p−1)2  +p2(p−1)(3p−10−2(−1p)−4(−3p))  +(p−p2)∑i=14|∑a=1p−1χ1i(a2(1−a))+2∑a=1p−1χ1i(a(1−a))−2|2  −p2(p−6)2 =3p4+O(p3).

So if 5 | *p* − 1, then combining (21) and (27) we can deduce the asymptotic formula

(28) ∑m=1p|∑a=1p−1e(ma5+nap)|4 =1+∑m=1p−1|∑a=1p−1e(ma5+nap)|4=3p3+O(p2).

Now our theorem follows from (25) and (28).

## Conflict of Interests

The authors declare that there is no conflict of interests regarding the publication of this paper.
